# Capability for arsenic mobilization in groundwater is distributed across broad phylogenetic lineages

**DOI:** 10.1371/journal.pone.0221694

**Published:** 2019-09-06

**Authors:** Robert E. Danczak, Michael D. Johnston, Chris Kenah, Michael Slattery, Michael J. Wilkins

**Affiliations:** 1 Department of Microbiology, Ohio State University, Columbus, OH, United States of America; 2 School of Earth Sciences, Ohio State University, Columbus, OH, United States of America; 3 Ohio Environmental Protection Agency, Columbus, OH, United States of America; 4 Department of Soil and Crop Sciences, Colorado State University, Fort Collins, CO, United States of America; Universidade Nova de Lisboa, PORTUGAL

## Abstract

Despite the importance of microbial activity in mobilizing arsenic in groundwater aquifers, the phylogenetic distribution of contributing microbial metabolisms is understudied. Groundwater samples from Ohio aquifers were analyzed using metagenomic sequencing to identify functional potential that could drive arsenic cycling, and revealed mechanisms for direct (i.e., *Ars* system) and indirect (i.e., iron reduction) arsenic mobilization in all samples, despite differing geochemical conditions. Analyses of 194 metagenome-assembled genomes (MAGs) revealed widespread functionality related to arsenic mobilization throughout the bacterial tree of life. While *arsB* and *arsC* genes (components of an arsenic resistance system) were found in diverse lineages with no apparent phylogenetic bias, putative *aioA* genes (aerobic arsenite oxidase) were predominantly identified in *Methylocystaceae* MAGs. Both previously described and undescribed respiratory arsenate reduction potential via *arrA* was detected in Betaproteobacteria, Deltaproteobacteria, and Nitrospirae MAGs, whereas sulfate reduction potential was primarily limited to members of the Deltaproteobacteria and Nitrospirae. Lastly, iron reduction potential was detected in the Ignavibacteria, Deltaproteobacteria, and Nitrospirae. These results expand the phylogenetic distribution of taxa that may play roles in arsenic mobilization in subsurface systems. Specifically, the Nitrospirae are a much more functionally diverse group than previously assumed and may play key biogeochemical roles in arsenic-contaminated ecosystems.

## Introduction

Arsenic contamination of groundwater is a pressing health issue throughout the world [1]. For example, elevated arsenic concentrations pose a threat to over 40 million people in Bangladesh and eastern India alone, particular those living in Bangladesh [2,3]. Meanwhile in the US, a recent study revealed that 20 of 37 principal aquifers contained arsenic concentrations above the maximum contaminant level of 10 μg L^-1^, affecting approximately 2.1 million [4,5]. Arsenic can occur in a range of redox states (-3, 0, +3, and +5), with mobile arsenite (As^3+^) and arsenate (As^5+^) the most common [6] in groundwater systems [7]. In this context, arsenite is more soluble and affected by sorption than arsenate [6] and presents a greater risk for human consumption.

The mobility of arsenic in these aquifer systems is at least partly mediated by microbial metabolism, via both direct and indirect mechanisms. Bacteria from diverse phylogenetic lineages feature resistance mechanisms that reduce and transport arsenic, such as the one encoded by the *ars* operon, where *arsB* is a membrane bound efflux pump and *arsC* is a cytoplasmic arsenate reductase [8–11]. Alternatively, a narrower phylogenetic distribution of microorganisms can participate in dissimilatory arsenic reduction via the *arr* system, utilizing arsenate as a terminal electron acceptor and converting it to arsenite [8–11]. Bacteria can also immobilize arsenic using arsenite oxidation mechanisms, such as the anaerobic (*arx*) and aerobic (*aio*) oxidase systems. Arsenic is further affected by the biogeochemical cycles of other inorganic groundwater constituents. The reductive dissolution of iron oxides through either direct microbial activity [12–14] or indirect biogenic sulfide production [15] can lead to release of adsorbed arsenic species. Although some previous studies have suggested that microbial sulfate reduction could immobilize arsenic through co-precipitation of sulfide-arsenic-iron species [16–18], other research has indicated increased arsenic mobilization associated with the formation of thioarsenic species that are more soluble and less likely to adsorb to iron minerals [19].

Despite our knowledge of microbial mechanisms that contribute to arsenic cycling, the phylogenetic distribution of this functional potential is less well understood. The recent application of metagenomic tools to shallow subsurface microbial populations revealed greater phylogenetic distribution of taxa involved in both nitrogen [20] and sulfur [21] cycling than was previously appreciated. Here, we applied similar tools to investigate the microbial potential for catalyzing arsenic transformations in shallow aquifers across three counties in central and southern Ohio (Athens, Greene, Licking). Geochemical and mineralogical analyses had previously suggested that groundwater in each county was at risk for elevated arsenic concentrations [7,22,23]. Leveraging metagenomic datasets, we investigated the relationship between the functional potential across diverse bacterial lineages, and elevated arsenic concentrations. Our data suggests that arsenic mobilization potential is found in groundwater ecosystems regardless of current geochemical conditions. Moreover, our results demonstrate that the potential for arsenic and iron mobilization is broadly distributed across the bacterial tree of life, with current analyses potentially missing many microbial groups capable of these transformations.

## Methods

### Sample collection

Groundwater samples were collected from three groundwater wells operated by the Ohio Department of Natural Resources (ODNR) in three different counties. These three wells are located within separate buried valley aquifers, consisting mostly of glacial sands and gravels, and some till. The observation wells were sampled on a quarterly basis over a two-year period from July 2014 to July 2016. One private drinking water well, located in a sand and gravel aquifer within a thick till sequence in western Licking County, was also sampled once in June 2016 [24].

Groundwater wells were sampled as previously reported [24,25]. Briefly, they were purged of more than 250 L of water to ensure that aquifer-derived water was being sampled (dedicated pumps were placed at the screened interval for the ODNR wells). Approximately 38L of post-purge groundwater was pumped sequentially through a 0.2 μm then 0.1 μm Supor PES Membrane Filter (Pall Corporation, NY, USA). Filters were then immediately flash frozen in an ethanol-dry ice bath, and kept on dry ice before being stored at -80°C at Ohio State University.

### DNA extraction, sequencing and processing

DNA was extracted from roughly a quarter of each 0.2 μm Supor PES membrane filter by using the Powersoil DNA Isolation Kit (MoBio Laboratories, Inc., Carlsbad, CA, USA). Final DNA concentrations were determined by using a Qubit Fluorometer (Invitrogen, Carlsbad, CA, USA).

Metagenomic data for 8 samples (filters from July 2014, Oct. 2014 and April 2016 for Greene and Athens, Oct 2014 for Licking, and the Licking–Private sample) was collected by shotgun sequencing on an Illumina HiSeq 2500 at the Genomics Shared Resource at the Ohio State University. Raw reads were trimmed and filtered based upon read quality using *sickle pe* with default parameters [26]. Resulting reads were subsequently assembled into larger contigs and then scaffolds using *idba_ud* with default parameters [27]. Assembly statistics are listed in **S1 Table**.

### Whole metagenome analysis

#### Arsenic and sulfur gene analysis

Each assembly was searched for a series of functional marker genes related to arsenic mobilization. All metagenomes were gene-called and translated using Prodigal with default parameters [28]. For genes encoding proteins which directly act upon arsenic (i.e. *arsB*, *arsC*, *aioA*, and *arrA*) [11], previously sequenced genes obtained from NCBI (**S1 File**) were used to create a BLAST database against which metagenomes could be searched [29]. Any gene that hit a previously identified sequence with an e-value of 1x10^-40^ or lower was considered a match. However, due to the interrelated nature of molybdopterin proteins, sequences identified as *arrA* or *aioA* underwent further analysis. Sequences potentially matching these two genes were subsequently aligned to other molybdopterin proteins (including NapA, DMSO reductase, etc.; **S1 File**) using MUSCLE with default parameters [30]. Alignments were then trimmed using Geneious v9.1.5 [31] to remove regions of at least 95% gaps and used to generate a RAxML tree with 100 bootstraps and an evolutionary model determined by ProtTest [32–34] (raxmlHPC-PTHREADS -f a -m ‘ProtTest-Output’ -n output -N 100 -p 1234 -s file.phy -x 1234 -T 20; ‘ProtTest-Output’ for arrA and aioA was WAG while all other tress were LG). Trees were visualized with R using ggtree [35] and only those sequences which fell into the respective *arrA* or *aioA* clades were analyzed further. The marker gene for sulfate reduction, *dsrD*, was identified in metagenomes using an HMM with trusted cutoff values generated by Anantharaman et al. (*hmmsearch—tbloout result*.*hres—noali—cut_tc -o result_hmm*.*txt dsrD*.*hmm input*.*faa*) [21,36].

#### Multiheme c-type cytochrome analysis

Multiheme c-type cytochromes (MHCs) required separate analyses for identification due to their numerous biochemical roles [37]. Potential MHC sequences were identified if a given amino acid sequence contained at least 3 CXXCH (Cys-X-X-Cys-His) motifs [37]. Potentially non-metal active MHCs were then removed through three primary steps. Firstly, the MHCs were annotated by comparing sequences to the KEGG, UniRef90, and InterproScan [38–42] databases using USEARCH [43] to scan for single and reverse best hit (RBH) results [24]. These annotations helped identify which cytochromes have a known function unassociated with metal cycling (i.e. NapC/NirT, NrfA, HAO, etc.). These sequences, along with seeded MHC sequences from *Geobacter* spp. and *Shewanella oneidiensis*, were clustered into a network by alignment score using EFI-EST [44,45] with a score cutoff of 67. These networks were then visualized using Cytoscape v3.4.0 [46] and parsed by hand in order remove hypothetical or misidentified proteins clustering with non-metal active proteins. Lastly, sequences were analyzed using PSORTb v3.0.2 [47] to localize encoded proteins within the cell, removing any protein destined for the cytoplasm only. Resulting protein and motif counts were plotted in R using the *ggplot2* package (v2.2.1) [48,49].

#### Gene abundance calculation

Functional gene abundances were determined by mapping trimmed reads from a metagenome to corresponding functional sequences using bowtie2 (bowtie—fast) [50]. These mapped reads were then normalized to the length of the gene and the number of mapped reads in the assembly in order to obtain a “reads per kilobase million” or “RPKM” metric. This measurement allows for cross sample comparisons in gene abundances. These calculations were performed in R and then plotted using the *ggplot2* package (v2.2.1) [48].

### Metagenomic binning and analyses

Scaffolds were binned using a combined binning approach called “DAS Tool” [51], which allows for the dereplication of bins generated through different bin generation strategies. Using the read mapping information obtained during the whole metagenome analyses, assembled scaffolds >2500 bp were binned using MetaBAT (*metabat—superspecific*) [52]. Assembled scaffolds >1000 bp were also binned using Maxbin [53]. Bins obtained from both algorithms were then processed using DAS Tool with default parameters [51]. Diversity of these bins was assessed by identifying ribosomal protein S3 (rps3) sequences using AMPHORA 2 [54]. Obtained S3 sequences were aligned to an rps3 database from Hug et al. [55] and used in tree generation according to the protocol outlined in the “Arsenic and sulfur gene analysis” section (LG determined with the added utilization of Gblocks to mask phylogenetically uninformative regions) [56]. The maximum-likelihood tree was visualized using ggtree in R [35], with major phylogenetic groups highlighted. Genome completeness and contamination were measured using CheckM [57]. Bin completion measurements can be found in **S2 Table**.

Only those bins which were of medium quality or greater based upon newly updated standards (i.e., > = 50% complete, <10% contaminated; 306 total) [58] were analyzed further. Each bin was annotated using the pipeline described above (i.e., USEARCH comparing sequences against KEGG, UniRef90, and InterproScan databases); resulting general metabolic characteristics were summarized. Bins were blasted against the specific genes of interest found in the whole metagenomes (*arsB*, *arsC*, *arrA*, *aioA*, *dsrD*, and MHCs) to find organisms potentially capable of arsenic mobilization. If a MAG of interest contained a sequence encoding a potential RuBisCO, an alignment followed by tree generation as described with *arrA* and *aioA* was performed with RuBisCO sequences from NCBI to assess whether it was type-I, -II, -III, or -IV.

Genome-based replication rates were approximated by performing an “iRep” analysis on genomes at least 75% complete and less than 3% contaminated [59]. Given that iRep requires genomes to be less than 2% contaminated, we have noted where these divergences occurred in the supplemental materials (**S3 Table**). The reason for this difference was to increase the representation of arsenic mobilizing functions in our analyses. Trimmed reads from each metagenome were mapped to corresponding bins using bowtie2 (*bowtie2—fast*) [50] with unmapped reads removed using shrinksam with default parameters [60]. The iRep command was then run with defaults and results were plotted in R using the *ggplot2* package (v2.2.1).

Approximate taxonomy was assigned by placement in the rps3 maximum-likelihood tree as well through an NCBI BLAST search [61]. Those genomes belonging to the Nitrospirae, Ignavibacteria, and *Methylocystaceae* were analyzed more deeply using 43-protein concatenated trees. Single copy genes (SCGs) utilized during the phylogenetic placement step in CheckM [57] were identified in all genomes from this study and those published on NCBI using HMMs obtained from PFAM (*hmmsearch -Z 26740544 -E 1e-20—tblout result*.*hres phylo*.*hmm input*.*faa*) [36,62]. These SCGs were then individually aligned, trimmed, and used in tree generation according to the method established in the “Arsenic and sulfur gene analysis” section, with an additional alignment concatenation step performed in Geneious v9.1.5 [31]. The trees were then visualized in FigTree v1.4.3 (http://tree.bio.ed.ac.uk/software/figtree/). An exhaustive literature search was performed to ascertain where throughout Bangladesh and eastern India these microorganisms were previously described. Studies were selected if they fell within the specified geographic region and whether they described, at some point, members of the *Methylocystaceae* or Nitrospirae (or sub-classifications).

Metagenomic read files, assemblies, bins, and protein files can be found on Cyverse (https://de.cyverse.org/de/). To access the files, users must create an account and log in. The following files are in the folder pathway “/iplant/home/danczakre/Ohio Groundwater Metagenomes”. Files are also available at the NCBI associated with bioproject number PRJNA512237.

### Map generation

R-compatible shapefiles for Bangladesh and India were downloaded from the Database of Global Administrative Areas (GADM) [63]. The shapefiles were imported in R using the *sp* package v1.3.1 [64], processed using the “fortify” command (*rgeos* package v0.3.28) [65], and plotted using the *ggplot2* package (v2.2.1) [48]. Points on the map were placed by hand based upon GPS coordinates.

## Results and discussion

### Groundwater microbiomes host functional potential for arsenic cycling, regardless of geochemical conditions

The geochemical conditions within three of these aquifers have been reported previously [25]. Briefly, the aquifer in Athens County was characterized by reducing conditions, and contained high concentrations of sulfate and dissolved iron. In contrast, samples from the Greene County aquifer were the most oxidizing, while the Licking aquifer featured elevated arsenic concentrations [25]. The fourth location, Licking–Private, had sulfate concentrations comparable to the Athens aquifer (151 mg/L), iron concentrations higher than the other Licking location (3.1 mg/L) and arsenic three times higher than the maximum contaminant limit (31 μg/L) (**S4 Table**).

**Fig 1:**
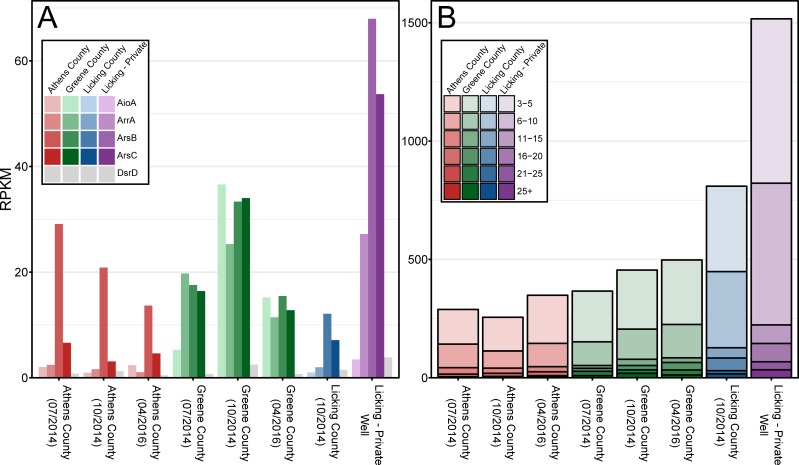
RPKM values for genes involved in potential direct or indirect arsenic mobilization. RPKM values for (A) arsenic mobilizing and sulfate reducing genes, and (B) c-type cytochromes across the 4 wells. Different colors represent different samples in both panels. In panel B, different shades of colors group sequences based upon similar CxxCH-motif counts. Licking–Private Well has the highest arsenic concentrations while Green has the lowest.

To investigate the metabolic potential for iron and arsenic cycling within these systems, normalized abundances of marker genes associated with arsenic (*arrA*, *arsBC*, *aioA*) and iron (multiheme c-type cytochromes; MHCs, *dsrD*) mobilization were compared across locations (**Fig 1**). The Licking–Private Well featured the greatest functional potential for both direct iron reduction and arsenic reduction, mirroring the surrounding geochemical conditions (**Fig 1**). Similarly, the genomic potential for arsenic oxidation was highest in the most oxidizing Greene County samples (**Fig 1**). However, the absence of correlations between genomic functional potential and geochemical conditions in the Athens and Licking aquifers suggest that measures of activity may provide additional insights into links between microbiomes and biogeochemical transformations. Similar inferences regarding the disconnect between functional potential and microbial activity have been made in other ecosystems [66–68]. This additionally supports previous research which noted that arsenic metabolism genes were found in rice paddies containing low concentrations of arsenic [69].

**Fig 2:**
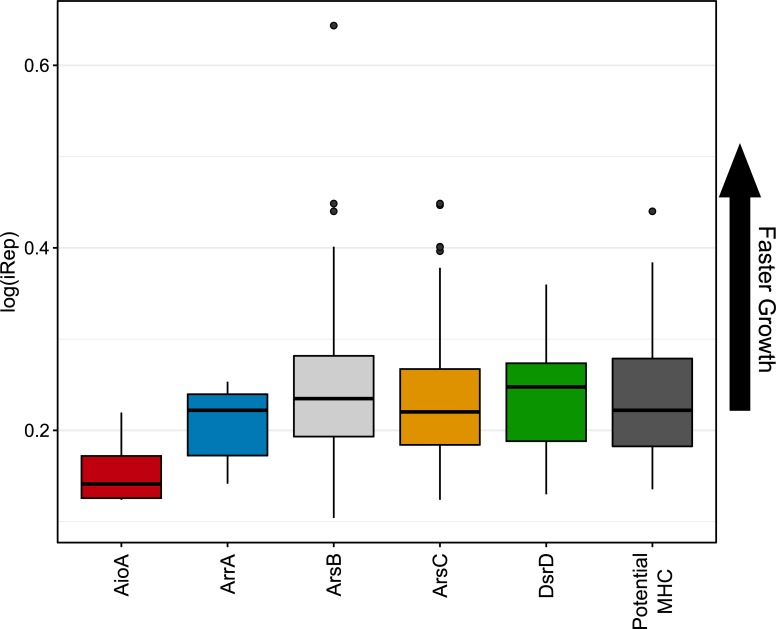
iRep values for genomes separated by functional potential. Higher log(iRep) values for the genomes containing the specified functional genes suggest faster growth. For example, those genomes which encoded putative AioA sequences had lower iRep values and replicated at a slower rate than those genomes encoding the other functions.

To obtain a proxy for microbial activity, average replications rates for metagenome-assembled genomes (MAGs) were calculated utilizing iRep (**Fig 2**) [59]. In brief, iRep measures variations in read coverage along a genome from the origin to the terminus to approximate replication rates [59]. While this metric does not directly relate to metabolic activity, it may help determine variations in replication rates between groups of microorganisms inferred to perform different functional roles in the environment. MAGs encoding the potential for iron, sulfate, and arsenate reduction featured similar, but variable, iRep values suggesting comparable overall rates of replication (**Fig 2**). In contrast, MAGs encoding the potential for arsenic oxidation (*aioA*) were all phylogenetically related to methanotrophs, and had potentially the slowest rates of replication, as inferred from the lowest iRep values (**Fig 2**). Given the absence of putative methanogens in this dataset, and the high oxidation-reduction potential measured in groundwater, slower inferred growth rates for methanotrophs may be related to electron donor availability.

### The metabolic potential for direct arsenic transformations exists throughout the bacterial tree of life

**Fig 3:**
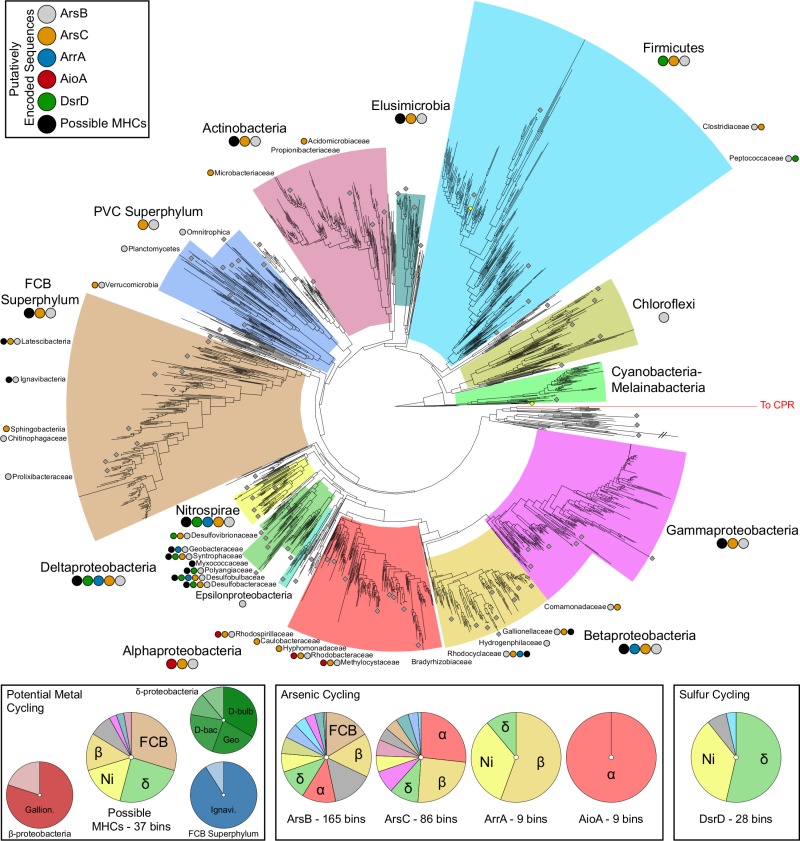
A maximum-likelihood tree generated from rps3 (rpsC) sequences. Each major lineage detected in this study is highlighted in a different color. Colored circles below each name indicate which of the examined functions were observed in MAGs belonging to each lineage. Gray circles at the end of branches indicate lineages observed in this study. In the lower portion of the figure, panels depict the frequency with which the putatively encoded functions occurred across MAGs belonging to the detected linages. For example, AioA is found exclusively in MAGs belonging to the Alphaproteobacteria.

To expand our understanding of metal-cycling microorganisms in shallow subsurface systems, MAGs were investigated for specific functions related to iron and arsenic transformations (**Fig 3**; **S2 File**). In total, 306 MAGs of medium or greater quality were recovered from 8 groundwater metagenomes (**S2 Table**). Of these MAGs, 194 were subsequently analyzed because they featured a ribosomal protein S3 sequence and at least one function of interest. Reflecting the conservation of the *ars* operon throughout archaea, bacteria, and eukaryotes, both *arsB* and *arsC*–arsenic detoxification genes–were broadly distributed throughout the bacterial tree of life and exhibited little phylogenetic bias within our dataset [9,11] (**Fig 3**).

**Fig 4:**
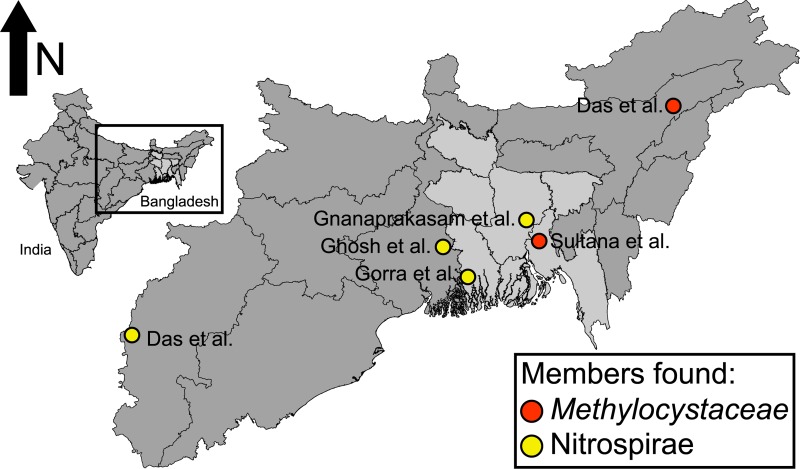
Biogeography throughout the India and Bangladesh. Map of eastern India and Bangladesh–areas historically affected by arsenic contamination–indicating the biogeographical distribution of bacteria related to the *Methylocystaceae* and Nitrospirae and the studies in which they were observed.

Contrastingly, the phylogenetic distribution of *aioA* was significantly more constrained, occurring only in genomes related to the *Methylocystaceae*, *Rhodosprillaceae*, and *Rhodobacteraceeae* families within the Alphaproteobacteria. Out of nine higher quality MAGs containing *aioA*, six genomes belonged to the *Methylocystaceae* and were most closely related to *Methylocystis rosea* SV97 and associated isolates, suggesting that these might constitute a new species within the genus. While *aioA* has been previously detected within *Methylocystis* sp. SC2, potential functionality has not been explored in significant detail. Based upon the analyses presented here, it appears that our MAGs feature metabolisms of previously characterized *Methylocystaceae* genomes, including methanotrophy via an encoded serine pathway as determined by the presence of methanol dehydrogenase and methane monooxygenase. Five MAGs appear to be missing a glycine hydroxymethyltransferase, however, and all are missing an alanine-glyoxylate transaminase. The *aioA* sequences within the *Methylocystaceae* appear to be most closely related to those found in the Order *Rhizobiales* (**S3 File, S1A Fig**), including the *aioA* found in *Rhizobium* sp. NT-26 which can obtain energy from arsenite oxidation [70]. Additionally, each genome appears to encode a putative pseudoazurin, a potential electron acceptor for the arsenite oxidase [70]. While these results suggest that the *Methylocystaceae* MAGs might be capable of conserving energy by oxidizing arsenite to arsenate, a role for these genes in canonical arsenic detoxification cannot be discounted. Regardless of energy conservation, members of this family have been found in other arsenic contaminated sites throughout the world, including numerous locations throughout Bangladesh [71,72] (**Fig 4**). Given their distribution throughout As-impacted sediments, members of the *Methylocystaceae* may play an under-appreciated role in arsenic cycling.

Nine medium or higher quality MAGs within the Betaproteobacteria, Deltaproteobacteria, and Nitrospirae contained putative *arrA* sequences (**Fig 3**). All five MAGs belonging to the Betaproteobacteria were members of the family *Rhodocyclaceae*, a functionally diverse and broadly distributed bacterial group [73]. The only Deltaproteobacteria MAG was a member of the *Geobacteraceae*. Lastly, the remaining three MAGs were members of the phylum Nitrospirae and were most closely related to genomes obtained from other subsurface environments [20,74,75]. Our results indicate that the organisms potentially capable of arsenic reduction might be more diverse than existing databases suggest; out of nine MAGs, only one belonged to a traditional arsenic reducing group (*Geobacteraceae*) (**Fig 3**). Particularly, both the *Rhodocyclaceae* and Nitrospirae represent groups that might be overlooked when considering only characterized arsenate reducers, and may represent a significant underestimation of arsenic mobilization potential throughout shallow aquifer systems due to their broad geographical distribution (**Fig 4**).

### Lineages throughout the tree of life have previously undescribed sulfate and iron reduction potential

Twenty-eight MAGs recovered in this study featured the potential to reduce sulfate to hydrogen sulfide. The majority (15) of genomes encoding this functional potential belonged to the Deltaproteobacteria (**Fig 3**), including members of the *Desulfobulbaceae*, *Desulfovibrionaceae*, *Desulfobacteraceae*, and *Syntrophaceae*. Additionally, a member of the family *Polyangiaceae* also displayed genomic features for this function. Although this group is not traditionally associated with sulfate reduction, this MAG encoded a potentially reductive *dsrABD* (**S4 File, S1B Fig**) and featured the potential to use a broad range of smaller carbon substrates as electron donors, including acetate, alcohols, and lactate, providing added genomic support for this function. Beyond the Deltaproteobacteria, the next abundant group of MAGs (10) inferred to perform sulfate reduction belonged to the phylum Nitrospirae. While traditionally considered to be nitrite oxidizers [76], increasing recent evidence has greatly expanded the functional potential of members within this phylum [21,77,78]. Again, these data suggest that the potential for sulfate reduction is present in phylogenetically broader groups than might be assumed by simply considering well-characterized isolates. The metabolic activity of these organisms could potentially drive indirect metal mobilization across diverse subsurface ecosystems.

**Fig 5:**
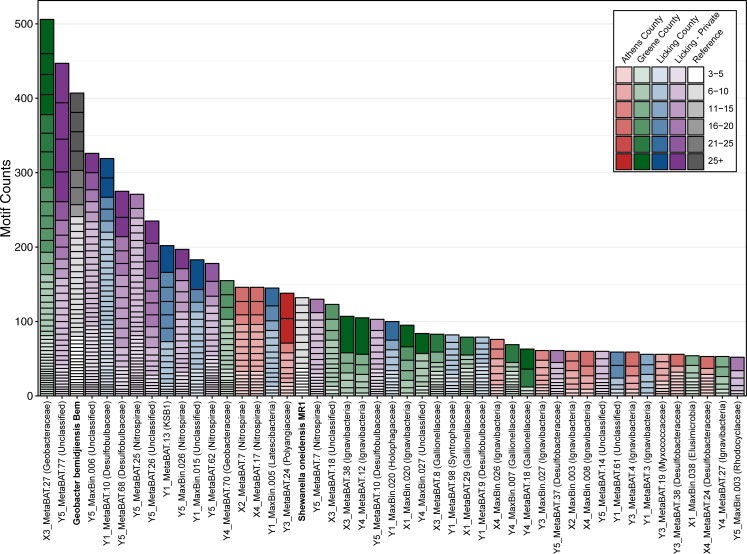
CxxCH-motif content for each MAG featuring over 50 motifs per genome. Each bar consists of small boxes which represent individual multiheme c-type cytochromes (MHCs) and are scaled based upon the number of CxxCH motifs within each sequence. Relative direct electron transfer potential of an organism can be approximated by the height of each bar. The phylogenetic relationship for each MAG is given in the parentheses along the x-axis and reference genomes of well-characterized iron reducing microorganisms (e.g., *G*. *bemidjiensis* Bem and *S*. *oneidensis* MR1) have bolded names and gray bars.

The reductive dissolution of iron oxides drives metal mobilization, with iron-reducing microorganisms using multi-heme c-type cytochromes (MHCs) as terminal reductases for the final step of electron transfer. Using the presence of putative metal-active MHCs as a screen for iron-reducing microorganisms, thirty-seven MAGs were selected for further analyses. (**Figs 3 and 5**). Nine of these MAGs were affiliated with the Deltaproteobacteria, including members of the *Desulfobacteraceae*, *Geobacteraceae*, and *Desulfobulbaceae* that are commonly inferred to catalyze iron reduction in the environment [79–81]. Moreover, many of these organisms encoded the capability to utilize shorter carbon compounds as electron donors via acetate kinases and alcohol dehydrogenases.

Beyond these traditional iron-reducing taxa, many MHC-encoding MAGs (10) belonged to the phylum Ignavibacteria within the FCB Superphylum. Some members of the Ignavibacteria, namely *Melioribacter roseus*, have previously been characterized as iron reducers [82,83], although their role in environmentally relevant metal cycling is less well understood [84]. Genome-level phylogeny of these Ignavibacteria indicated that eight of the ten were most closely related to *Melioribacter* (**S5 File**, **S1C Fig**). A closer examination of the metabolic potential in these organisms revealed many similarities with other Ignavibacteria and canonical iron reducers, such as the inferred ability to utilize organic acids (acetate, formate, and lactate). Moreover, each genome encoded a complete electron transport chain with all but 2 organisms exhibiting the potential to utilize diverse terminal electron acceptors including nitrite (reduced to ammonia via the NrfAH system) and nitrous oxide (reduced to nitrogen via NosZ). MHCs within the Ignavibacteria MAGs featured an average of 10 heme-binding motifs, placing them within the range of MHCs detected in other iron cycling microorganisms (**Fig 5**). In the absence of traditional autotrophic pathways associated with iron oxidizers, and the phylogenetic relationship to the known iron reducer *Melioribacter rosesus*, we infer that these Ignavibacteria MAGs are potentially capable of iron reduction. Although members of the Ignavibacteria appear to be broadly distributed in numerous groundwater ecosystems, relatively few seem to have been specifically detected in arsenic contaminated systems. We hypothesize that this may at least partly be due to mis-identification of these taxa as deeply branching members of the Chlorobi prior to their recent organization into a new phylum (Ignavibacteriae) [82]. Overall, these results suggest that members of the Ignavibacteria–beyond the characterized isolate *Melioribacter rosesus–*likely catalyze reductive dissolution of iron oxides, contributing to the mobilization of adsorbed metals in reducing environments.

Additional MAGs containing a high number of putative metal-active MHCs were distributed across the Betaproteobacteria and Nitrospirae. Within the Betaproteobacteria, four out of the five MAGs were members of the *Gallionellaceae*. Each of these MAGs featured chemolithoautotrophic metabolic potential, including an encoded RuBisCO, and were most closely related to other groundwater *Gallionellaceae* genomes, although BLAST results suggest that the nearest related isolates were the iron-oxidizing organisms *Siderooxydans lithotrophicus* and *Ferriphaselus amnicola* [85]. Together, these results indicate that the *Gallionellaceae* MAGs recovered here likely catalyze iron oxidation reactions. The functional potential encoded within the Nitrospirae MAGs is discussed in more detail below.

### The phylum Nitrospirae could play significant, previously undescribed roles in arsenic cycling

The phylum Nitrospirae contains the distantly-related genera *Leptospirillum*, *Thermodesulfovibirio*, and *Nitrospira*, and features a broad diversity of metabolic functionality [76,77,86–89]. Isolated members of the *Leptospirillum* have been characterized as chemolithoautotrophic iron-oxidizers, in contrast to the sulfate reducing capacity of the *Thermodesulfovibrio*, or the nitrite-oxidizing capabilities of the *Nitrospira* [76,77,86,90]. Additionally, a clade related to the *Thermodesulfovibrio* contains genes necessary for magnetotaxis via intracellular magnetite formation [91]. We were able to assemble fourteen medium quality or greater MAGs spanning the phylogenetic and functional diversity within the phylum. Moreover, we were able to uncover a novel potential function in arsenic cycling for the Nitrospirae, in addition to assigning a potentially greater role in iron cycling.

A 43-protein concatenated tree (**S6 File**, **S1D Fig**) revealed that the 13 Nitrospirae MAGs generated here are more closely related to genomes obtained from other subsurface environments than to isolated species, while one is related to Candidatus *Nitrospira nitrificans*. Metabolically, these organisms encoded respiratory functionality based upon the presence of oxidative phosphorylation genes, and contained a broad range of functions typical within the Nitrospirae. Firstly, two MAGs contained genes (e.g., *nxrA/B*) for nitrite-oxidizing capabilities traditionally characteristic of this phylum. Three MAGs encoded nitrate reductases via *napA*, three MAGs featured sequences for copper-containing nitrite reductases (e.g., *nirK*), and three MAGs encoded putative nitric oxide reductases (*norBC*). Evidence for nitrite reduction to ammonium was present in one MAG featuring the Nrf-system, and others encoded a distantly related ammonia forming nitrite reductase. Additionally, ten MAGs encoded the inferred functional potential for sulfate reduction via the presence of reductive-type *dsrABD* genes [21].

MHC profiles similar to known iron-reducers, *Geobacter bemidjiensis* and *Shewanella oneidensis*, were found in six MAGs, suggesting that members outside of the genus *Leptospirllum* may be capable of iron cycling (**Fig 5**). The presence of MHCs with a broad range of CxxCH motifs (up to 49 within one protein) suggests that members of the Nitrospirae may be capable of direct election transfer to or from iron oxides. Due to similar enzymatic machinery and associated genomic features, disentangling iron reduction from iron oxidation is challenging in the absence of microbial isolates. Some evidence suggests that at least two of these Nitrospirae MAGs may perform chemolithoautotrophy due to the presence of the large subunit of a type-II RubisCO (**S7 File**, **S1E Fig**). While this mechanism is found in the genus *Leptospirillum*, these MAGs appear to be unrelated, suggesting that this functionality might be widespread throughout the phylum. In contrast, the absence of RuBisCO-based autotrophic machinery suggests that four Nitrospirae MAGs may perform iron reduction. These MAGs are potentially capable of utilizing ethanol or formate as electron donors via an alcohol dehydrogenase and pyruvate formate lyase / Wood-Ljungdahl pathway, respectively. Additionally, two MAGs may be capable of acetate utilization via an ADP-forming acetyl-CoA synthetase; propionate and butyrate are unlikely substrates, however. While these observations are based upon genomic inferences, results suggest that members of the Nitrospirae may play additional, previously unrecognized roles in iron cycling.

Lastly, the capacity for respiratory arsenate reduction via a putatively encoded *arrA* was found in three of the high quality Nitrospirae MAGs representing a possible metabolic expansion for this phylum. Broadening our analyses to 185 other published genomes, *arrA* sequences were found in only eleven other Nitrospirae beyond the three described here. All 14 *arrA* sequences appear to be most closely related to those sequences found in Deltaproteobacteria genomes, suggesting a potentially shared evolutionary history or horizontal gene transfer (**S8 File**, **S1F Fig**). Geographically, members of the Nitrospirae appear to be constituents of microbial communities in many ecosystems, including those featuring elevated arsenic concentrations [92–95] (**Fig 4**). While much of this evidence is based upon 16S rRNA gene data, numerous Nitrospirae are members of groundwater bacterial communities obtained from Bangladesh and might participate in direct arsenic transformations. These results further suggest that the capability for arsenate reduction is broadly distributed and likely exists in other under-sampled lineages. Moreover, the Nitrospirae might represent a previously unidentified group of organisms capable of widespread arsenic mobilization.

## Conclusions

Arsenic-contaminated groundwater is a pressing issue throughout the world, although the relationship between contributing geochemical conditions and microbial communities is underexplored. Here we present evidence that the functional potential for arsenic mobilization is both geographically and phylogenetically widespread (**Figs 1 and 3**). In the groundwater samples studied here, moderate changes in environmental conditions could potentially stimulate the activity of arsenic-cycling microorganisms. For example, the onset of more reducing conditions in the Greene County location studied here could lead to increasing concentrations of mobile arsenic species through the activity of *arsC* and *arrA* genes that catalyze arsenate reduction.

Furthermore, our research demonstrates the importance of investigating microbial community members beyond “canonical” iron or arsenic cycling organisms. The *Methylocystaceae* are a family consisting of methanotrophs but some members appear to encode an *aioA*, allowing them to potentially oxidize arsenite (**Fig 3**). The phylum Ignavibacteria appears to contain a greater diversity of members encoding potential genes necessary for iron reduction, a function previously described only within the genus *Melioribacter*. Lastly, members of the Nitrospirae are much more functionally diverse than previously thought, with new groups potentially capable of iron reduction, iron oxidation, and arsenate reduction. Moreover, members of the *Methylocystaceae* and Nitrospirae are detected in locations affected by elevated groundwater arsenic concentrations and may help explain the mobilization potential missed by tracking standard organisms. Although these functions are inferred from genomic data alone, these results highlight the importance of looking beyond exclusively isolation-based and phylogenetic assumptions. By obtaining a more complete understanding of the diverse functional potential encoded within subsurface microbiomes, we can begin to combine isolation-based techniques with arsenic mobilization studies in an attempt to better address this ongoing crisis.

## Supporting information

S1 Fig PDF containing all trees generated within this study: A) AioA tree, B) DsrAB tree, C) Ignavibacteria concatenated tree, D) Nitrospirae concatenated tree, E) RuBisCO tree, and F) ArrA tree. Red labels indicate sequences from this study, while blue are reference sequences.
(PDF)

S1 File FASTA file containing all arsenic-related sequences from NCBI used in this study. (FASTA)

S2 File Raw maximum-likelihood *rpsC* tree run with 100 bootstraps. (TRE)

S3 File Maximum-likelihood *AioA* and other molybdopterin-protein tree run with 100 bootstraps. (TRE)

S4 File Concatenated *dsrAB* maximum-likelihood tree run with 100 bootstraps. (TRE)

S5 File Concatenated maximum-likelihood tree generated from 16 ribosomal proteins for all published Ignavibacteria genomes from NCBI. (TRE)

S6 File Concatenated maximum-likelihood tree generated from 16 ribosomal proteins for all published Nitrospirae genomes from NCBI. (TRE)

S7 File Maximum-likelihood tree for RuBisCO sequences run with 100 bootstraps. (TRE)

S8 File Maximum-likelihood tree for *ArrA* sequences run with 100 bootstraps. (TRE)

S1 Table Table of assembly statistics for each of the 8 metagenomes. (XLSX)

S2 Table Table of bin information, including phylogeny, completion, contamination, and other relevant information. (XLSX)

S3 Table Table of unaltered iRep results. (CSV)

S4 Table Geochemistry results for historical samples and those used in this study (highlighted in green). (XLSX)
