# Supplementary figures and images for: Capability for arsenic mobilization in groundwater is distributed across broad phylogenetic lineages
Source: PLoS One. 2019 Sep 6;14(9):e0221694. doi: 10.1371/journal.pone.0221694 (PMC6730927; doi:10.1371/journal.pone.0221694)

# A. AioA Tree

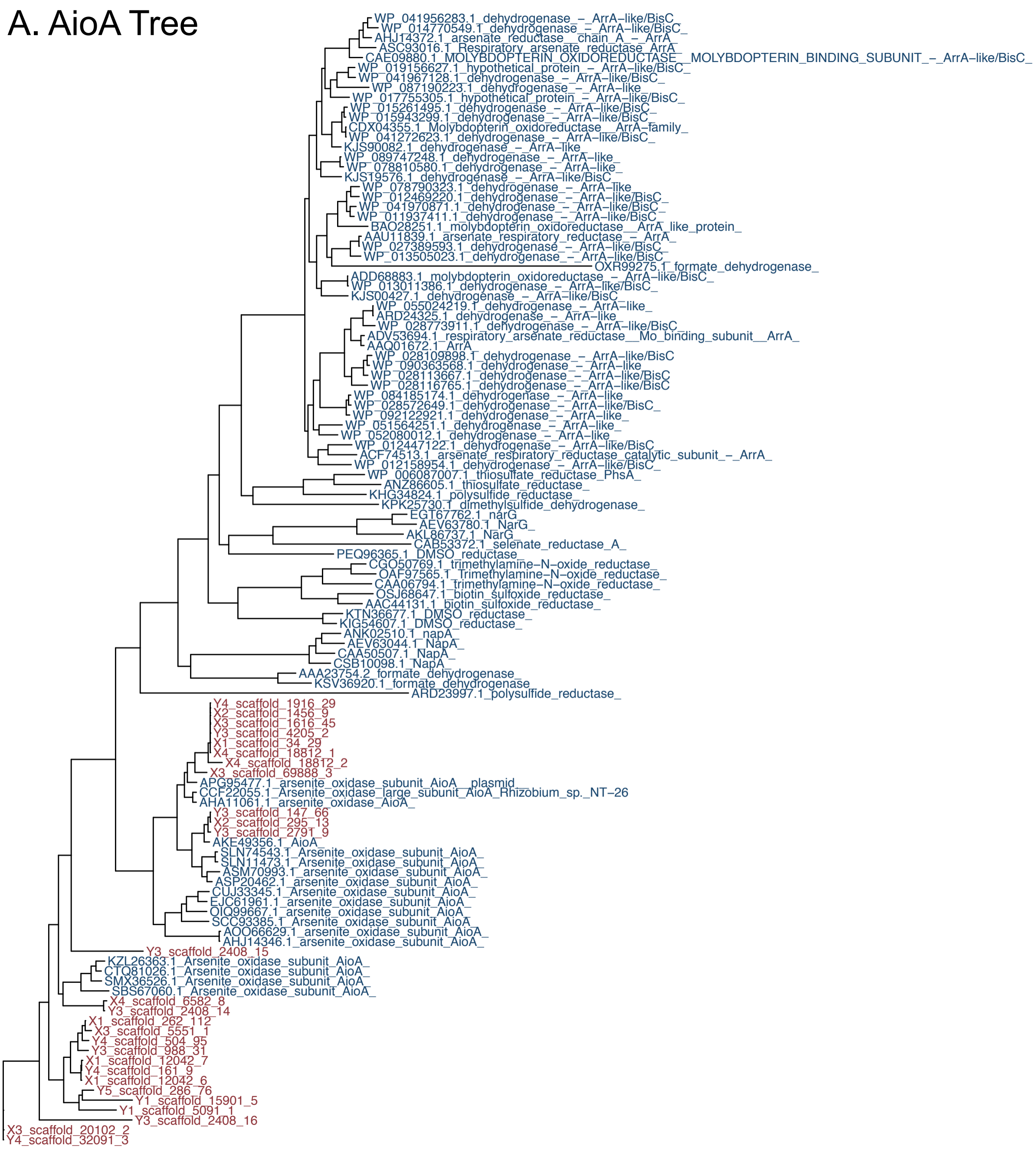

# B. DsrAB Tree

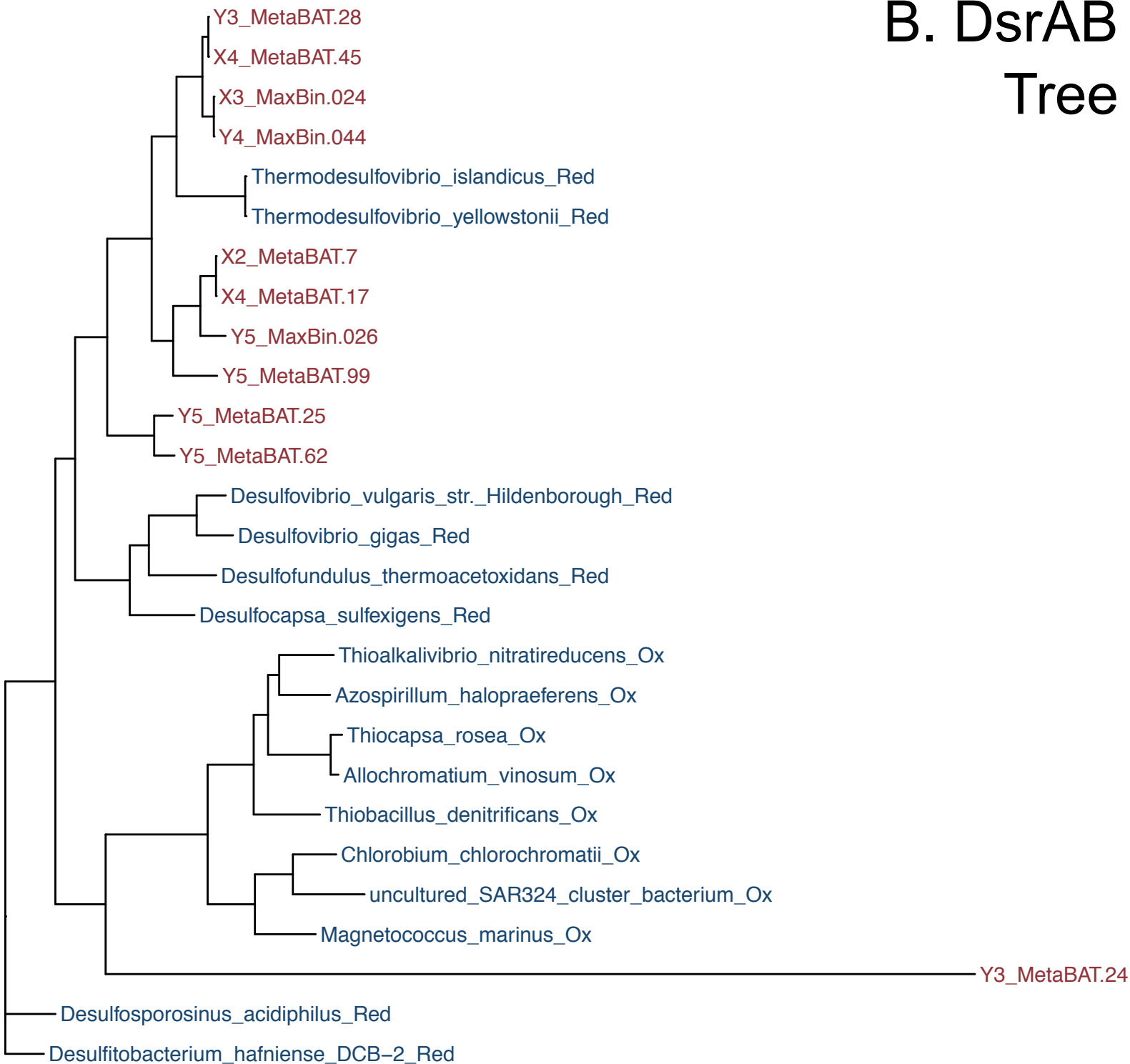

# C. Ignavibacteria Tree

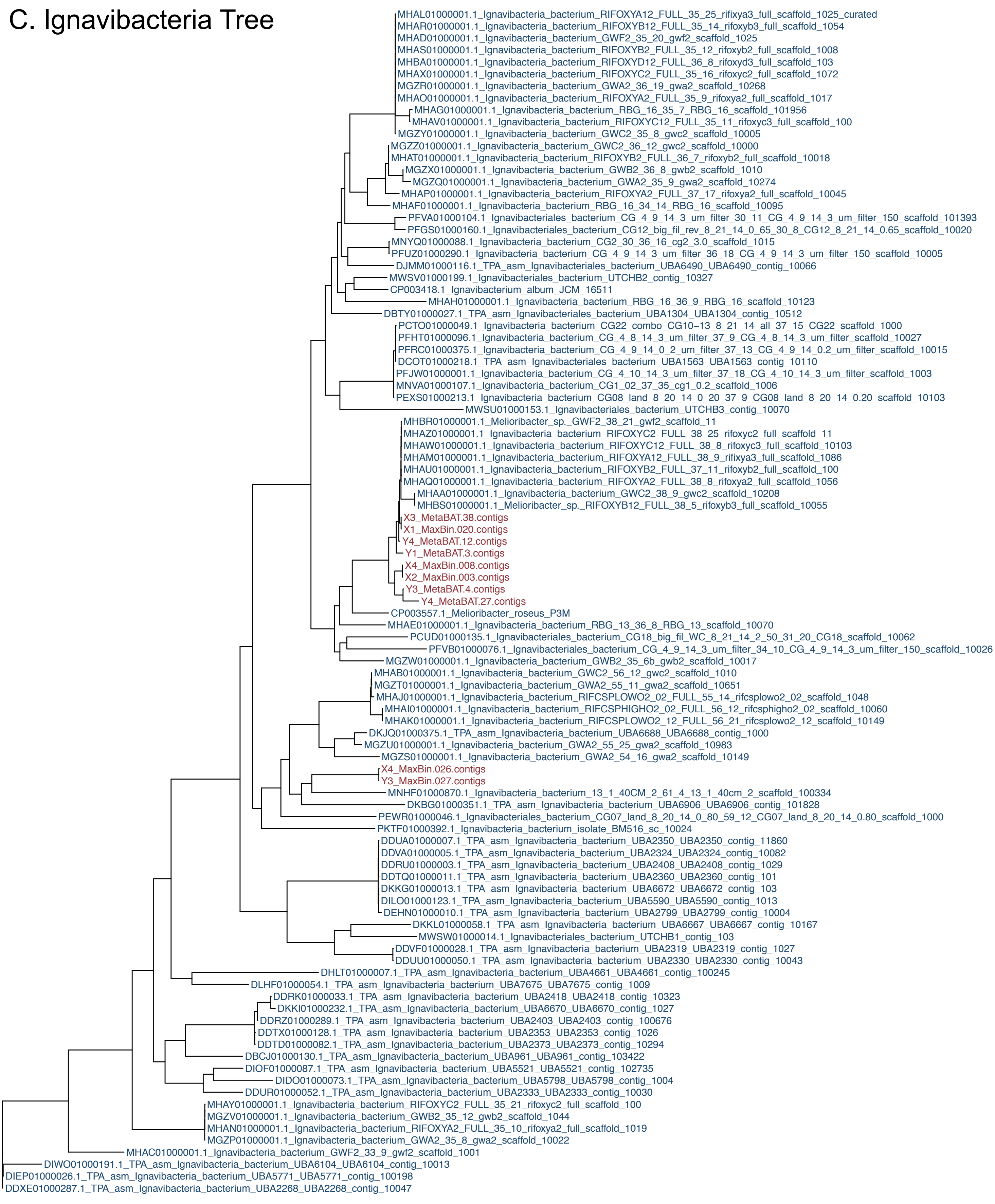

D. Nitrospirae Tree

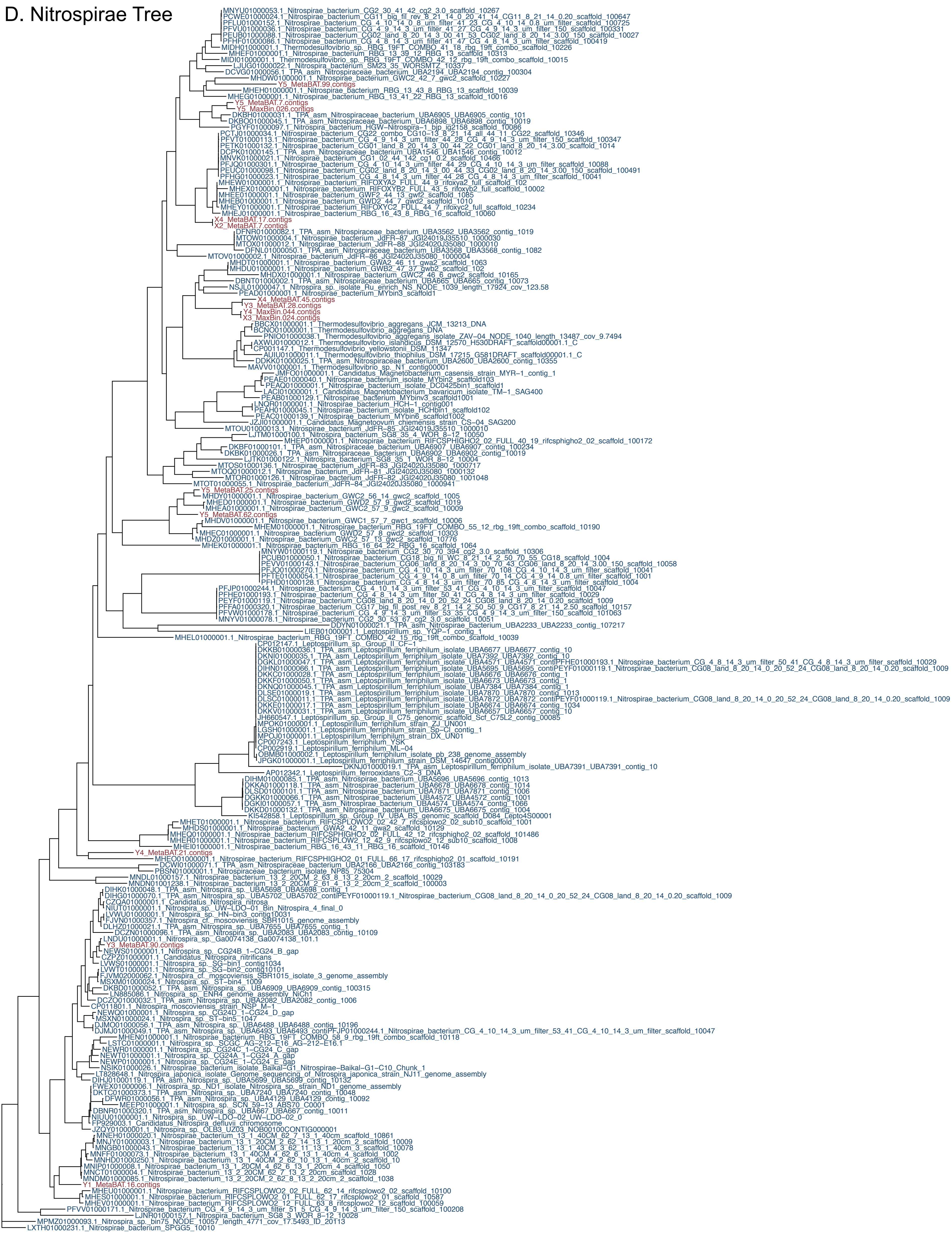

# E. RuBisCO Tree

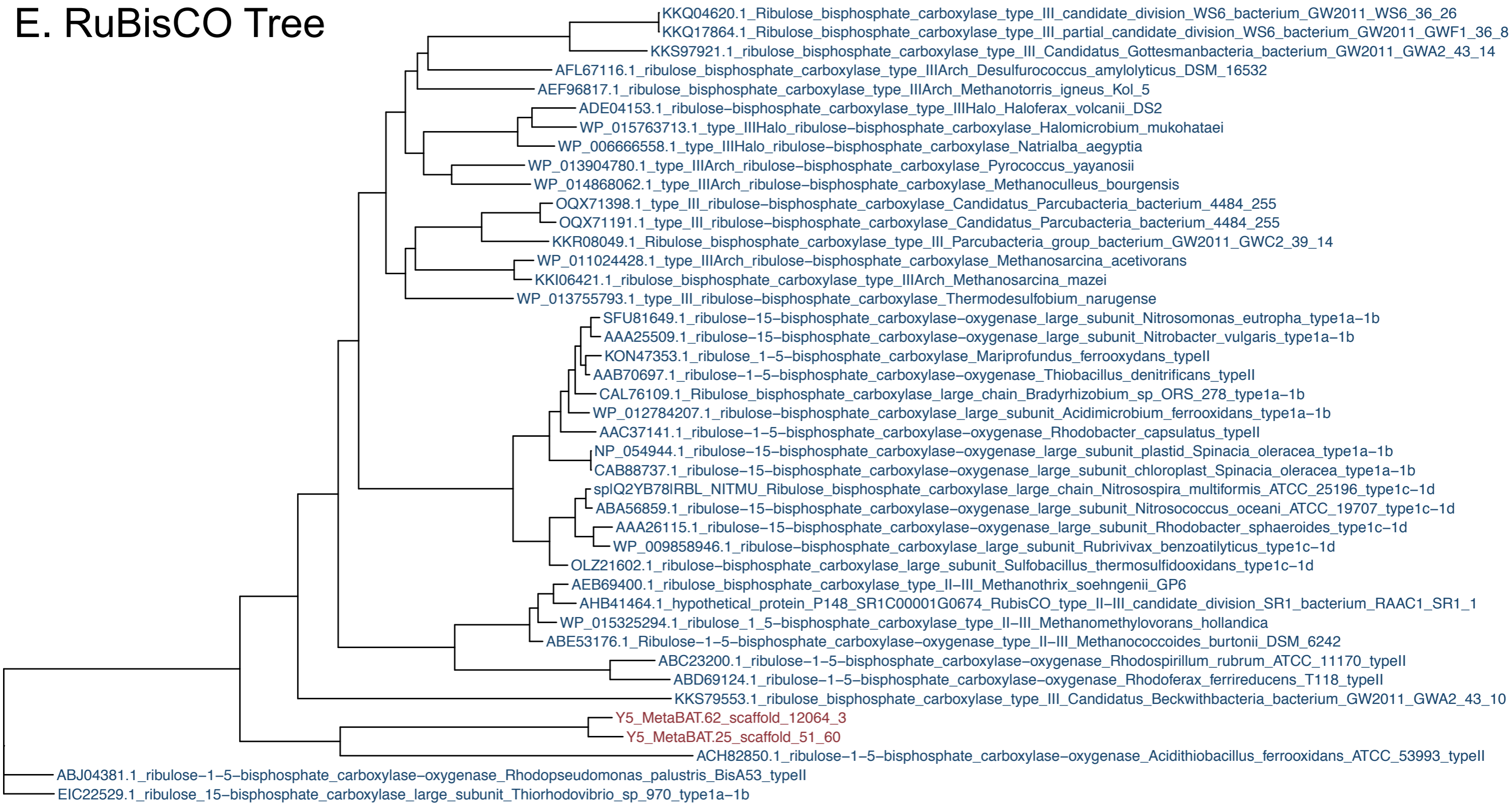

## F. ArrA Tree

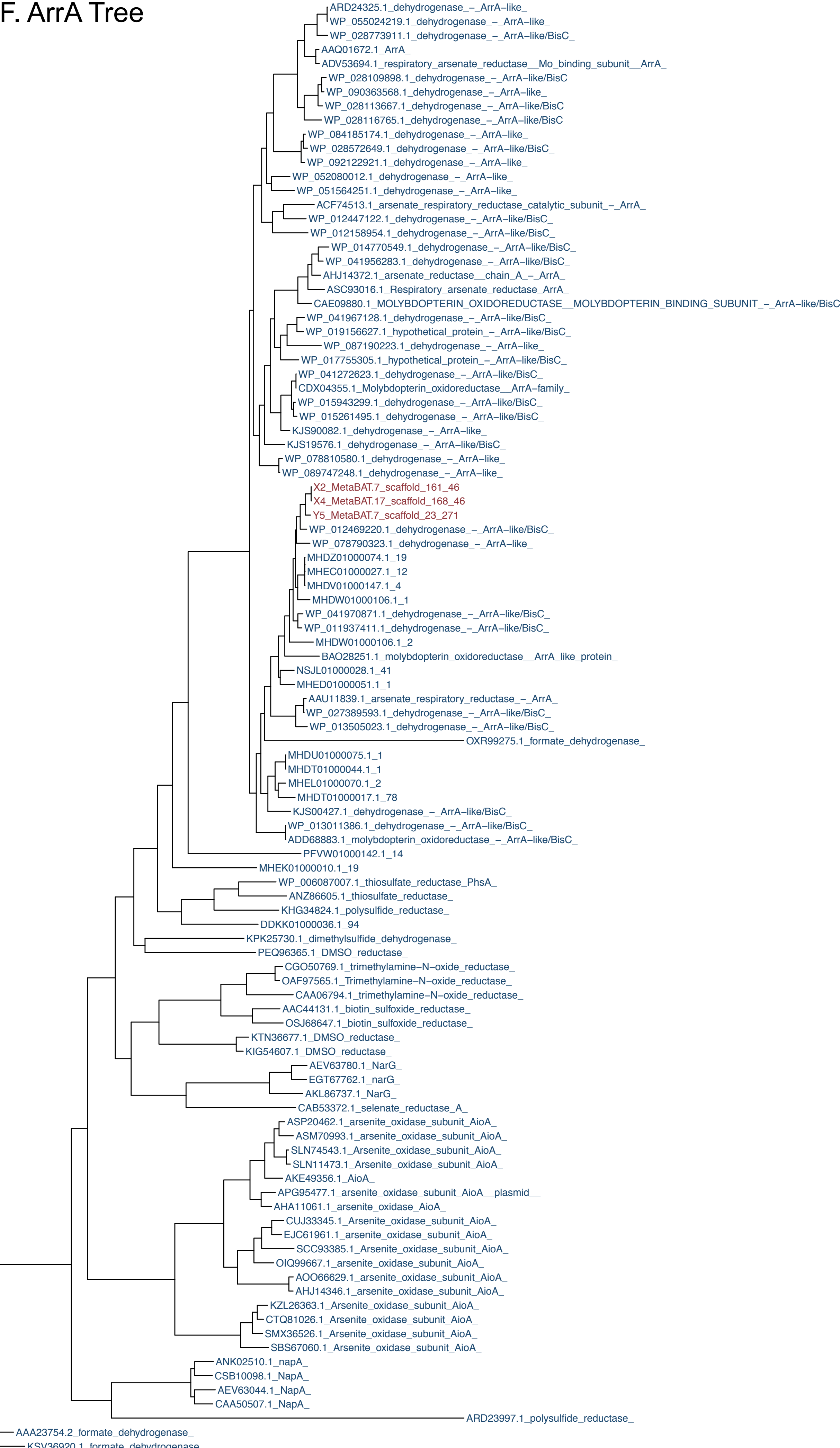

Supplement: S1 Fig — PDF containing all trees generated within this study: A) AioA tree, B) DsrAB tree, C) Ignavibacteria concatenated tree, D) Nitrospirae concatenated tree, E) RuBisCO tree, and F) ArrA tree. Red labels indicate sequences from this study, while blue are reference sequences. (PDF) [file pone.0221694.s001.pdf]
